# Circadian Rhythm Protein Bmal1 Modulates Cartilage Gene Expression in Temporomandibular Joint Osteoarthritis *via* the MAPK/ERK Pathway

**DOI:** 10.3389/fphar.2020.527744

**Published:** 2020-09-18

**Authors:** Guokun Chen, Haoming Zhao, Shixing Ma, Lei Chen, Gaoyi Wu, Yong Zhu, Jie Zhu, Chuan Ma, Huaqiang Zhao

**Affiliations:** ^1^ Department of Oral and Maxillofacial Surgery, School and Hospital of Stomatology, Cheeloo College of Medicine, Shandong University and Shandong Key Laboratory of Oral Tissue Regeneration and Shandong Engineering Laboratory for Dental Materials and Oral Tissue Regeneration, Jinan, China; ^2^ Department of Orthodontics, School and Hospital of Stomatology, Cheeloo College of Medicine, Shandong University and Shandong Key Laboratory of Oral Tissue Regeneration and Shandong Engineering Laboratory for Dental Materials and Oral Tissue Regeneration, Jinan, China; ^3^ Department of Plastic Surgery, Jinan Airong Plastic Surgery Hospital, Jinan, China

**Keywords:** circadian rhythm disturbance, osteoarthritis, temporomandibular joint, BMAL-1, cartilage, interleukin-6, MAPK/ERK pathway

## Abstract

The purpose of this study was to elucidate the role of the circadian gene Bmal1 in human cartilage and its crosstalk with the MAPK/ERK signaling pathway in temporomandibular joint osteoarthritis (TMJ-OA). We verified the periodical variation of the circadian gene Bmal1 and then established a modified multiple platform method (MMPM) to induce circadian rhythm disturbance leading to TMJ-OA. IL-6, p-ERK, and Bmal1 mRNA and protein expression levels were assessed by real-time RT-PCR and immunohistochemistry. Chondrocytes were treated with an ERK inhibitor (U0126), siRNA and plasmid targeting Bmal1 under IL-6 simulation; then, the cells were subjected to Western blotting to analyze the relationship between Bmal1 and the MAPK/ERK pathway. We found that sleep rhythm disturbance can downregulate the circadian gene BMAL-1 and improve phosphorylated ERK (p-ERK) and IL-6 levels. Furthermore, Bmal1 siRNA transfection was sufficient to improve the p-ERK level and aggravate OA-like gene expression changes under IL-6 stimulation. Bmal1 overexpression relieved the alterations induced by IL-6, which was consistent with the effect of U0126 (an ERK inhibitor). However, we also found that BMAL1 upregulation can decrease ERK phosphorylation, whereas ERK downregulation did not change BMAL1 expression. Collectively, this study provides new insight into the regulatory mechanism that links chondrocyte BMAL1 to cartilage maintenance and repair in TMJ-OA *via* the MAPK/ERK pathway and suggests that circadian rhythm disruption is a risk factor for TMJ-OA.

## Introduction

Mammals produced endogenous circadian clocks to adapt to the changes in the day and night of the earth’s rotation, which are organized in a cycle of about 24 h (Mohawk et al., 2012; Welz and Benitah, 2020). Circadian rhythm is an inherent survival instinct of organisms that enables them to predict and prepare for predictable environmental changes caused by the earth’s daily rotation. The circadian rhythm disturbance caused by insomnia and shift work can lead to many sub-health conditions and diseases, such as obesity, diabetes, osteoarthritis, cardiovascular disease, and so on (Downton et al., 2019; Rijo-Ferreira and Takahashi, 2019; Albrecht, 2020). The circadian clock consists of central clock pacemaker and peripheral clock receptor. Its central pacemaker located in the suprachiasmatic nucleus of the hypothalamus. They receive time information from external timing signals, mainly light: dark (LD) cycle changes (Welsh et al., 2010; Mohawk and Takahashi, 2011).

For this reason, mammalian cells contain rhythm genes that interact in the intracellular oscillatory transcriptional network and regulate the expression of many other genes that are essential to cell physiology and metabolism. As a central rhythm pacemaker, SCN causes changes in cortisol in the blood through the HPA axis, and this circadian rhythm changes help carry the clock signal of the central clock pacemaker to the peripheral clock receptor. In peripheral tissues, the circadian rhythm of cells is maintained by a feedback loop that automatically regulates transcription and translation with two branches: the positive regulatory branch of the CLOCK and BMAL-1, and the negative regulatory branch composed of PERs and CRYs (Patke et al., 2020). Circadian rhythm gene BMAL-1 is an important part of mammalian clock gene regulatory network. It is a sensitive point in the network because it is the only gene in a mouse model where single gene knockout causes arrhythmias at the molecular and behavioral levels (Bunger et al., 2000). BMAL1 has been shown to be important in bone metabolism because the bone mass of BMAL1 KO mice is lower than that of wild-type mice (Song et al., 2018). Recently, researchers have discovered the role of circadian rhythm in the musculoskeletal system (Song et al., 2018). Recent studies have shown that chronic circadian rhythm disturbance interference is closely related to the development of osteoarthritis-like lesions of the knee joint in mouse, thus revealing circadian rhythm disturbance as a new risk factor for OA (Ranjan et al., 2015).

Under normal circadian rhythm, chondrocytes keep the circadian rhythm stable, and the expression of rhythm-related proteins is normal, which can regulate the dynamic balance between “resting cartilage repair” and “exercise cartilage wear” (Pérez-García et al., 2019). However, under the condition of osteoarthritis, the matrix balance is destroyed, which leads to the gradual loss of cartilage matrix, the clonal expansion of cells, and finally leads to the apoptosis of chondrocytes. The up-regulation of cartilage matrix catabolism and/or down-regulation of cartilage matrix synthesis lead to matrix balance disorder and cartilage degradation (Mobasheri and Batt, 2016). The production of inflammatory cytokines (i.e.,IL-1,IL-6,TNF-α) and chondrocyte matrix-degrading enzymes (i.e., MMPs and ADAMTs) increased, while the production of synthetic enzymes and anti-inflammatory factors (i.e., COL2,aggrecan) decreased, resulting in degeneration of articular cartilage (Mehana et al., 2019). Apoptosis of articular chondrocytes will eventually lead to complete loss of articular cartilage and exposure of subchondral bone, which will lead to wear and tear due to unbuffered direct contact between bones, resulting in pain and limited joint movement (Xia et al., 2014). A series of cartilage matrix catabolism mediators and cartilage matrix synthesis metabolism mediators play a key role in the occurrence and development of articular cartilage homeostasis and OA. Among many representative mediators, interleukin-6 (IL-6) is one of the best-known compounds characterized by omnidirectional interactions in processes, especially in osteoarthritis occurring in the human body. Inflammatory factor IL-6 is considered to play an important role in the development of osteoarthritis, which strongly activates the immune system and enhances joint inflammation. Recent studies have found that in cases of TMJ-OA involves internal derangement and bony changes, the synovial fluid of these joints contains interleukin-1b (IL-1b), a major pro-inflammatory cytokine, with higher levels of IL-1b than normal TMJ. IL-1b upregulates interleukin 6 (IL-6) and interleukin-8 (IL-8), two other cytokines found in the synovial fluid of TMJ (Kaneyama et al., 2005). Our previous studies have shown that after circadian rhythm disturbance, MAPK/ERK signaling pathway is activated and the phosphorylation level of ERK is enhanced; in addition, the increase of MMPs and ADAMTs downstream induced by ERK leads to cartilage decomposition leading to pathological changes of temporomandibular joint synovium and condylar cartilage (Ma et al., 2014). The components of circadian oscillator can directly interact with MAPK/ERK signal pathway, and ERK-mediated phosphorylation of downstream related proteins may play an important role in maintaining biological circadian rhythm (Sanada et al., 2002; Goldsmith and Bell-Pedersen, 2013).

In this study, we used the modified multiple platform method (MMPM) to disrupt the circadian rhythm of rats to explorbe the relationship between circadian rhythm disturbance and TMJ-OA. Through the study of this *in vivo* rats model, we provide direct evidence that circadian rhythm disturbance play a key role in the development of TMJ-OA. We observed lower BMAL-1 expression and higher P-ERK/MMPs/ADAMTS expression in the circadian rhythm disruption group than in the control group and circadian rhythm recovery group. Then, we elucidated the roles of BMAL-1/P-ERK by directly targeting BMAL-1 expression, and disrupting BMAL-1 expression lead to the downregulation of P-ERK in chondrocytes. The deregulation of the expression of circadian gene BMAL-1 may increase the expression of MMP and ADAMT by activating ERK/MAPK pathway.

## Method and Materials

### Animal Experimental Design

One hundred eighty male 8-week-old Wistar rats (weight, 220 ± 20 g) were purchased from the Laboratory Animal and medicine Center of Shandong University (Jinan, China) and were raised in the same laboratory. The animals were housed in cages in a temperature-controlled room at 24°C under a 12:12-h light-dark cycle and received food and water without restriction. The animals were adjusted to laboratory conditions for 3 days without any manual intervention. Then, the Wistar rats were adapted to the animal model for environmental circadian rhythm disruption for 30 min per day for four successive days before the start of the experiment.

The rats were then divided randomly into three groups (60 in each group): the control (CON) group, the circadian rhythm disturbance (CRD) group, and the circadian rhythm recovery (REC) group. The three groups were divided equally into three subgroups (n=20 each) according to the different time points at which the rats were sacrificed (4, 6, and 8 weeks). The CRD and REC rats were placed on small platforms during the procedure at the same time, as described in the subsequent sections of this article. At the scheduled time, the rats in the CRD group were sacrificed, while the rats in the REC group were allowed to stay in cages for 1 week to adjust their circadian rhythm back to normal. Rats in the CON group were housed in cages in the same room.

### TMJ Tissue Collection

All rats were sacrificed after 4, 6, and 8 weeks according to their subgroups with an overdose of pentobarbital sodium, and their bilateral TMJs were excised rapidly. The TMJs were dissected, and 5 rats’ bilateral TMJs were selected randomly from each group for hematoxylin and eosin (HE) staining and immunohistochemistry (IHC). These isolated TMJs were fixed in 10% buffered paraformaldehyde. Additionally, the remaining 15 rat joints were selected to determine the BMAL-1, ERK, P-ERK, MMPs, ADAMTS, and COL2 expression by Western blotting and real-time quantitative polymerase chain reaction (PCR). These TMJ specimens were dissected and flash frozen with liquid nitrogen. Two pieces of mandibular condylar cartilage from each rat were treated as one sample to guarantee that enough protein and mRNA was available for the analysis.

### Animal Model for Environmental CRD

**Figure 1:**
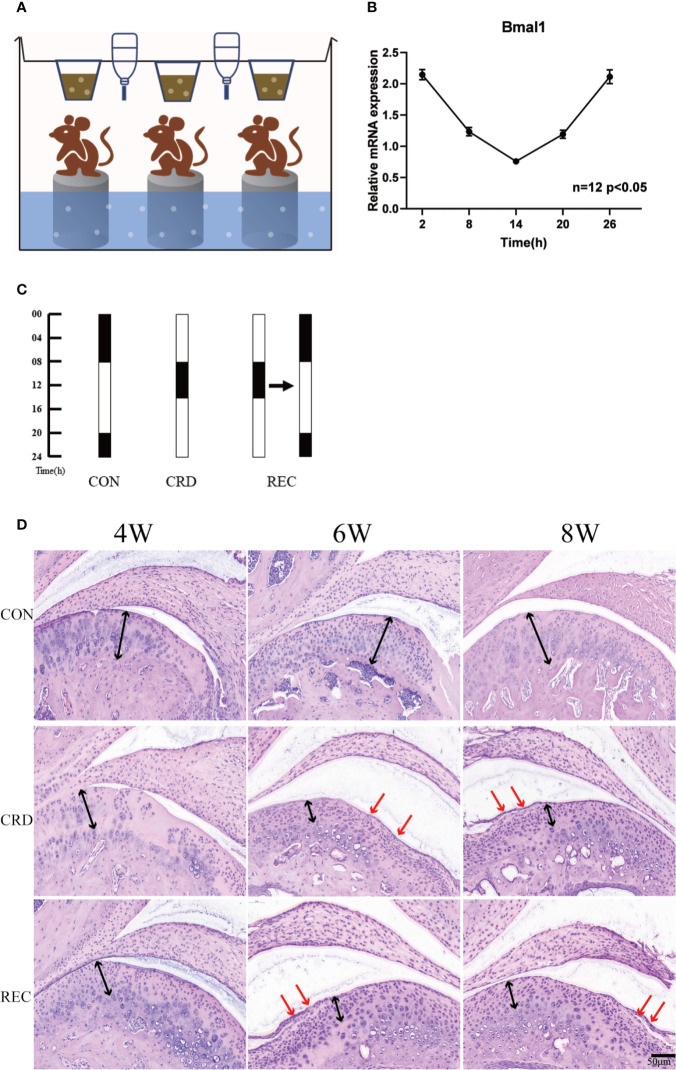
Circadian rhythm disturbance (CRD) induced experimental TMJ-OA in rats. **(A)** CRD created by a modified multiplatform method (n=3 rats per group). **(B)** Circadian expression of *bmal1* genes in the spleen. **(C)** Grouping arrangement and light:dark (LD) cycle arrangement of animal experiments. **(D)** Rat TMJ sections subjected to hematoxylin and eosin (HE) staining. The black arrows indicate chondrocyte disarrangement, cluster formation and cell-free areas in the CRD and SEC groups. The red arrows indicate cartilage loss and surface integrity reductions. All experiments were performed in triplicate, and the results are expressed as the mean ± SD. The P value is written in the figure corner. Scale bar: 50 μm.

The modified multiple platform method (MMPM) was selected to induce CRD in this study (Machado et al., 2004). As shown in **Figure 1**, the rats were placed inside a tiled glass water tank made of organic glass (145.0 cm long × 44.0 cm wide × 45.0 cm high) containing 28 narrow circular platforms (6.5 cm in diameter). The narrow platforms were set 12 cm apart from each other so that the rats could only stand on them. During the experiment, the two tanks were filled with water until there was approximately 1 cm from the surface of the water to the platform. Then, 20 rats were placed in each tank so that they could move around freely by jumping from one platform to another among the 28 platforms. Finally, the water tank was covered with iron mesh, and food and water were placed on the iron mesh. When the rats reached the paradoxical phase of sleep, they were awakened when their faces touched the water as a result of muscle atonia. Thus, CRD was achieved by depriving the rats of paradoxical sleep. The rats were placed on the platforms for 18 h per day (14:00—8:00+1 day) under controlled room (24 ± 2°C) and water (18 ± 2°C) temperatures. After each 18-h circadian rhythm disruption, the animals could sleep in their individual home cages for 6 h (beginning at 8:00). The water in the tank was changed daily throughout the CRD period.

### Histological Staining and Immunohistochemistry

Following sacrifice, condyles and articular disks from TMJs were dissected aseptically and fixed in 4% buffered paraformaldehyde for 24 h, decalcified with 10% EDTA at 4°C for 4 weeks and then embedded in paraffin. The samples were cut into 5-μm-thick sagittal sections and stained with HE. The sections were then evaluated for degenerative changes by three blinded graders according to a modified scoring system for mouse articular cartilage, with a score of zero representing unaltered cartilage and six representing severe OA. The scores for each section from three blinded graders were averaged, resulting in total scores between 0 and 6 (**Table 1**) (Bomsta et al., 2006). Immunohistochemical staining was carried out using the standard streptavidin-peroxidase (S-P) method. For IHC, after 3% hydrogen peroxide treatment and antigen retrieval, all tissue sections were blocked at room temperature with 5% bovine serum albumin (BSA) for 30 min and then incubated with primary antibodies overnight at 4°C. A secondary antibody, biotinylated anti-rabbit IgG, was applied for 30 min at room temperature (Vector Laboratories). The percent of positively stained cells per field (positive cell %) was determined for an average of three fields in each slide. The immunohistochemistry results were analyzed by image capture under a microscope at 400× magnification and quantification with Image-Pro^®^ Plus software (Ranjan et al., 2015).

**Table 1:** Cartilage erosion scoring system used to evaluate articular cartilage of mice.

Feature	Score
Smooth noneroded cartilage	0
Rough noneroded cartilage	1
Superficial fibrillation	2
Separation of uncalcified from calcified cartilage	3
Erosion of uncalcified cartilage only	4
Erosion extending into calcified cartilage	5
Erosion down to the subchondral bone	6

### RNA Extraction and Analysis by Real-Time Quantitative PCR

For RNA extraction, TMJ tissues were harvested and cut into small pieces (< 2 mm^2^ × 2 mm^2^). The pieces were frozen immediately in liquid nitrogen and then ground into powder. Total RNA was extracted using TRIzol reagent (Invitrogen) according to the manufacturer’s protocol. For RT-PCR, we used a SYBR Two Step RT-qPCR Kit (Takaba, USA, Code No. RR037A) following the manufacturer’s instructions. The cycles were as follows: initial denaturation at 95°C for 30 s, followed by 40 amplification cycles of 95°C for 5 s and 60°C for 30 s. The resulting values were quantified using the comparative cycle threshold method, and the samples were normalized to GAPDH. Quantification of the relative expression levels was determined by the 2^-ΔΔCT^ method. The primers for measuring mRNA are described in **Supplement Table 1**.

### Chondrocyte Isolation and Culture

Three-week-old Wistar rats were sacrificed with an overdose of pentobarbital sodium, and their bilateral TMJs were excised rapidly. First-passage chondrocytes were used for the subsequent experiments. The chondrocytes were cultured in DMEM containing 10% fetal bovine serum. Once reaching 80% confluence, the chondrocytes were cultured and plated onto 6-well plates for further study. After 3 days of culture when the 6-well plates reached 80% confluence, the chondrocytes were transfected with an siRNA and plasmid against BMAL-1-11 under IL-6 simulation.

### Small Interfering RNA (siRNA) and Plasmid Targeting BMAL-1 for Transfection Under IL-6 Simulation

siRNA targeting BMAL-1 and a BMAL-1 overexpression plasmid were purchased from RiboBio Biotechnology Inc. First-passage chondrocytes were transfected with siRNA or plasmid with Lipofectamine 2000 (Invitrogen) according to the manufacturer’s instructions. After transfection, the cells were seeded on a 6-well plate and cultured for 8 h. To induce catabolic stress, cells were cultured with 10 ng/μl IL-6 for another 12 h after transfection.

### Protein Sample Preparation and Western Blotting

Cell and tissue lysates were prepared using modified RIPA buffer. Total protein concentrations were determined by a bicinchoninic acid (BCA) protein assay (Beyotime, catalog P0012). Protein solutions (approximately 15 μl) were resolved by 10% SDS-polyacrylamide gel electrophoresis and transferred to polyvinylidene difluoride (PVDF) membranes (Millipore, catalog IPVH00010). The PVDF membranes with the proteins were blocked in 5% non-fat milk at room temperature for 2 h. Then, the membranes were incubated with primary antibodies, including anti-BMAL-1 (1:1000, Abcam, catalog ab231793), anti-Erk1/2 (1:1000, Cell Signaling, catalog #4695), anti-phospho-ERK1/2 (1:1000, Cell Signaling, catalog #4370), anti-ADAMTS5 (1:1000, Bioss, catalog bs3573R), anti-MMP3 (1:1000, Servicebio, catalog GB11131), anti-MMP13 (1:1000, Abcam, catalog ab39012), anti-COL2α1 (1:1000, Sigma, catalog SAB4500366), and anti-β-actin (1:2000, Servicebio, catalog GB11001). All primary antibodies were diluted with 5% TBST buffer (with 0.1% Tween 20) and incubated overnight at 4°C. After washing with TBST buffer (with 0.1% Tween 20) 3 times, the membranes were incubated for 2 h with secondary horseradish peroxidase-conjugated anti-mouse (1:10,000, Servicebio, catalog GB23301), and anti-rabbit (1:10,000, Servicebio, catalog GB23303) antibodies. After washing with TBST, the target proteins were detected on the membranes with an ECL detection system (Millipore, catalog MA01821). Western band images were captured digitally, and the intensity of the bands (pixels/band) was determined in arbitrary optical density units using ImageJ densitometry analysis software.

### Intra-Articular Adenovirus Injection

Adenoviruses expressing mouse Ad-NC and Ad-*bmal1* plasmids were purchased from RiboBio Biotechnology, Inc. Rat TMJ articular chondrocyte cells were cultured for 3 days to reach 80% confluence and then infected with adenovirus for 2 h. For IA adenovirus injection, Ad-NC-plasmid and Ad-*bmal1*-plasmid (1 × 10^9^ PFUs in a total volume of 10 μl) were injected into the TMJs of mice once per week for 4, 6, and 8 weeks in the CON, CRD, and REC groups.

### Alcian Blue Staining

Alcian blue (Solarbio, catalog G2542, PH2.5) was used in this experiment. Primary cultured cells were plated at a density of 8–9 × 10^4^ per well in 6-well plates. At the end of the indicated culture period, the cells were washed with ice-cold PBS 3 times, fixed at room temperature with 37% buffered formalin for 5 min, and rinsed with 0.1 N HCl. The cells were then stained at room temperature with Alcian blue for 30 min. After washing with PBS buffer, the staining results were captured by imaging under a microscope with 200× magnification and quantified with Image-Pro^®^ Plus software.

### Statistical Analysis

Statistical analyses were performed using GraphPad Prism software (GraphPad Software, Inc.). In the case of data with a normal distribution and/or equal variances, significant differences between two and more than three groups were determined by a two-tailed Student’s t test and one-way ANOVA followed by Bonferroni’s *post hoc* comparison test, respectively. P values lower than 0.05 were considered to be statistically significant. The results are presented as the mean SEM from at least three independent experiments.

## Results

### The TMJ-OA Rat Model Was Successfully Established by CRD

We created a rat model of CRD with the MMPM (**Figure 1A**). We quantified the expression profile of clock gene Bmal1 in rats under normal circadian rhythm. We found that Bmal1 gene expression was lower in the light cycle and higher in the dark cycle. These findings suggest that the sleep rhythm can be disrupted by depriving rats of sleep at night (**Figure 1B**). In the control group (CON), the normal appearance of rat condylar cartilage was determined by HE staining; the surface was smooth, and mature chondrocytes were evenly distributed in the cartilage, including the fibrocartilage layer and proliferating cell layer. Four of the cell layers and the mast cell layer showed no obvious histopathological changes (**Figure 1C**). The CRD group showed OA-like histopathological changes beginning in the 4^th^ week. For example, loss of cartilage surface integrity, reductions in chondrocytes and cell sequence disorders, and formation of chondrocyte clusters and acellular regions were observed (**Figure 1C**). With prolonged sleep deprivation times, we revealed that some of the 8-week samples had osteophyte-like structures, which proved that OA-like pathological changes gradually increased over time. In the circadian rhythm recovery group (REC) group, OA-like histopathological changes, such as irregular cartilage surfaces, cell sequence disorders and cell clusters, were also observed, but they were milder and less common than those in the CRD group. The chondral scores in the CRD group were significantly increased over time (CRD6/CRD4 = 2.34, P = 0.013; CRD8/CRD4 = 3.86, P = 0.019). The three time points in the CON group were 0. The CRD scores of the REC group also increased significantly with time (P <0.01), but the scores at 4 and 6 weeks were significantly lower than those in the CRD group. We compared the CRD group with the REC group and found that the early ratio was significantly higher than the late stage ratio (CRD4/REC4 = 4.3, CRD6/REC6 = 3.1, CRD8/REC8 = 1.0724, P < 0.05). These results demonstrate that sleep deprivation can cause OA-like lesions in the rat TMJ and may be reversible at an early stage.

### The Relationship Between BMAL-1-1/IL-6/P-ERK and TMJ-OA

**Figure 2:**
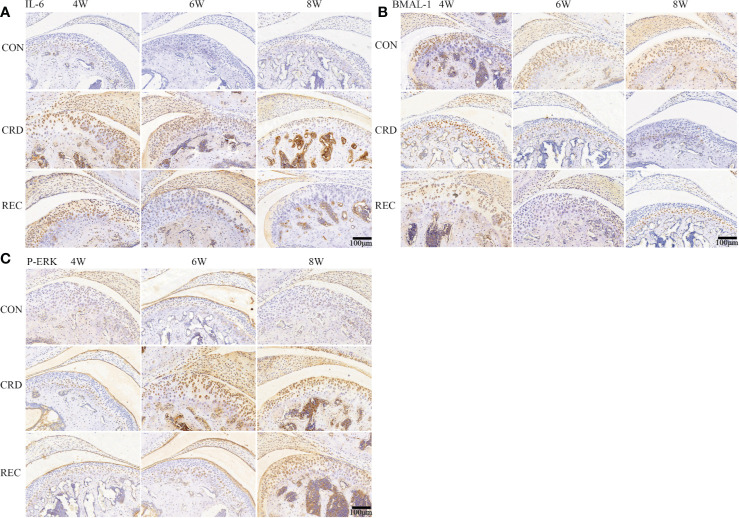
IL-6, BMAL-1, and P-ERK protein expression in rat condylar cartilage. Representative images of immunohistochemistry staining for IL-6 **(A)**, BMAL-1 **(B)**, and P-ERK **(C)** in the CON, CRD, and REC groups. All experiments were performed in triplicate, and the results are expressed as the mean ± SD. Scale bar: 100 μm.

**Figure 3:**
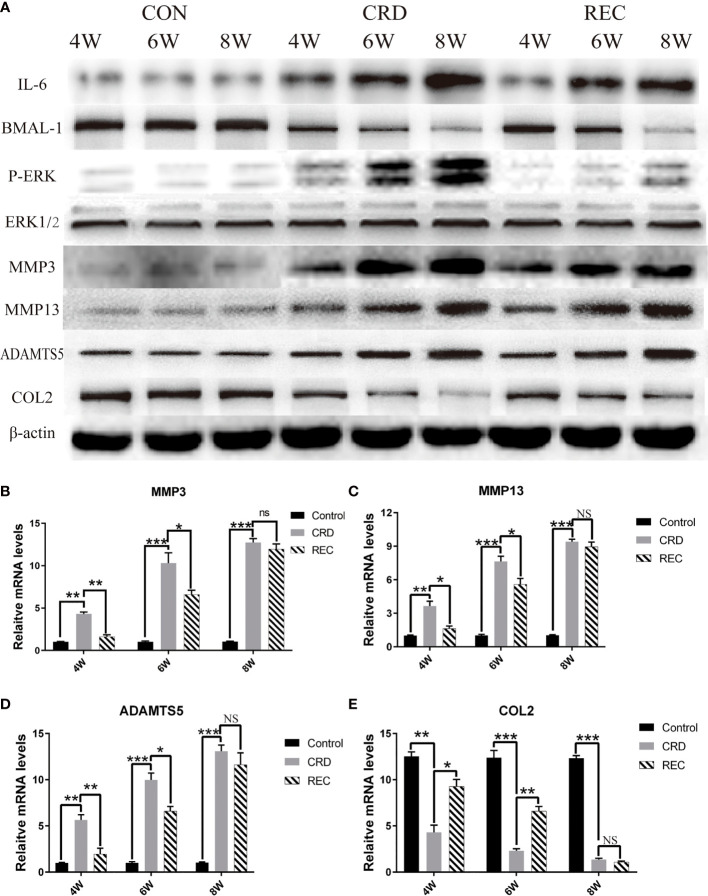
Expression trends for IL-6/P-ERK/BMAL-1/MMP3/MMP13/ADAMTS5/COL2 in the CON, CRD, and REC groups. **(A)** Western blot results for IL-6/P-ERK/BMAL-1/MMP3/MMP13/ADAMTS5/COL2. **(B–E**) RT-qPCR analysis of MMP3/MMP13/ADAMTS5/COL2. All experiments were performed in triplicate, and the results are expressed as the mean ± SD. *P < 0.05; **P < 0.01, ***P < 0.005. NS, not significant.

The immunohistochemical staining results for the control group indicated that the fibrocartilage cell layer showed almost no IL-6 and P-ERK expression. In the CRD group, IL-6 and P-ERK were positively expressed after 4 weeks of sleep deprivation, and IL-6 and P-ERK expression increased with prolonged sleep deprivation (**Figures 2A, C**). Contrary to the IL-6 expression trend, BMAL-1 was significantly decreased upon sleep deprivation for 4 weeks and continued to decrease with time (**Figure 2B**). In the REC4 and REC6 groups, we found a significant reversal of P-ERK and BMAL-1 expression levels. This result is also consistent with the HE and Western blotting results (**Figures 1C** and **3A**), confirming that early OA-like changes may be reversible at both the histological and protein expression levels. The reason for the above phenomenon is most likely caused by a change in the expression of the rhythm protein BMAL-1. The expression of the representative inflammatory factor IL-6 remained higher in the CRD4 and REC4 groups and was significantly decreased in the CRD4 and REC8 groups. This indicates that inflammatory factors are highly expressed in early OA and have high expression levels in serum and intra-articular synovial fluid around the joint, which is a characteristic manifestation of early OA. In contrast, after osteophyte formation due to the OA-like changes, the expression levels of IL-6 and other inflammatory factors are downregulated after recovery.

Western blotting showed that the expression trends for IL-6/P-ERK/BMAL-1 were similar to those shown by immunohistochemistry (**Figure 3A**). Indistinguishably, we also found that MMP3/13/ADAMTS5 expression in the CRD group was similar to that of IL-6 and P-ERK and increased with prolonged sleep deprivation times (**Figure 3A**). In the REC group, MMP3/13/ADAMTS5 expression at the mRNA and protein levels was ablated (**Figures 3A–D**). COL2 is an important constituent protein in the ECM of cartilage. We found that COL2 changes with the expression of BMAL-1, so BMAL-1 may be involved in regulating chondrocyte ECM formation (**Figures 3A, E**). MMPs and ADAMTS5 act as matrix-degrading enzymes, and their upregulation indicates large-scale degradation of ECM proteins such as COL2, which leads to cell sequence disorder and cell-free region formation. We found that BMAL-1 and ERK may regulate the expression of metalloproteinases.

### The Regulatory Relationship Between BMAL-1/P-ERK and IL-6

**Figure 4:**
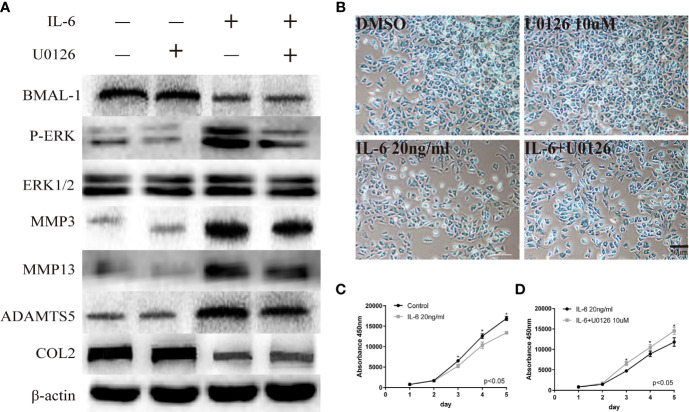
Regulatory relationship between BMAL-1/P-ERK and IL-6. **(A)** Western blot assays demonstrate the relationship between BMAL-1, P-ERK, MMP3, MMP9, MMP13, and COL2, while the expression levels of ERK1/2 and β-actin were the same in the cartilage tissues of the control group, CRD group and REC group. **(B)** Alcian blue staining results for MCCs stimulated with 20 nM or 10 µM U0126 and 20 ng/ml IL-6 for 12 h. **(C**, **D)** A CCK-8 assay was used to examine the cell proliferation of each group and the effects of u0126; the absorbance was measured at 450 nm. All experiments were performed in triplicate, and the results are expressed as the mean ±SD. The P value is written in the figure corner. Scale bar: 50 mm. *P < 0.05.

We used U0126, a specific inhibitor of phosphorylated components in MAPK/ERK signaling pathway, to verify the relationship between phosphorylated ERK and BMAL-1. U0126 was added to rat mandibular condylar chondrocytes (MCCs) to reduce P-ERK expression. Simultaneously, ERK/P-ERK, BMAL-1, TMJ-OA-related phenotype protein-MMP3/13, ADAMTS5, and COL2 were detected by Western blotting. We found that inhibiting ERK expression in the presence of IL-6 resulted in decreased expression of P-ERK and downstream MMP3/13 and ADAMTS5 and increased expression of the matrix protein COL2 (**Figure 4A**). However, BMAL-1 expression was not affected. Inhibiting ERK phosphorylation can inhibit the secretion of chondrocyte matrix-degrading enzymes but cannot influence the expression of BMAL-1. Similarly, Alcian blue staining and CCK-8 assays showed that IL-6 significantly inhibited chondrocyte proliferation and mesochondrium production (presented in **Figures 4B, C**), and U0126 blocked the effects of IL-6 on chondrocyte proliferation (**Figures 4B, D**).

### BMAL-1 Regulates TMJ-OA In Vitro *via* the MAPK/ERK Signaling Pathway

**Figure 5:**
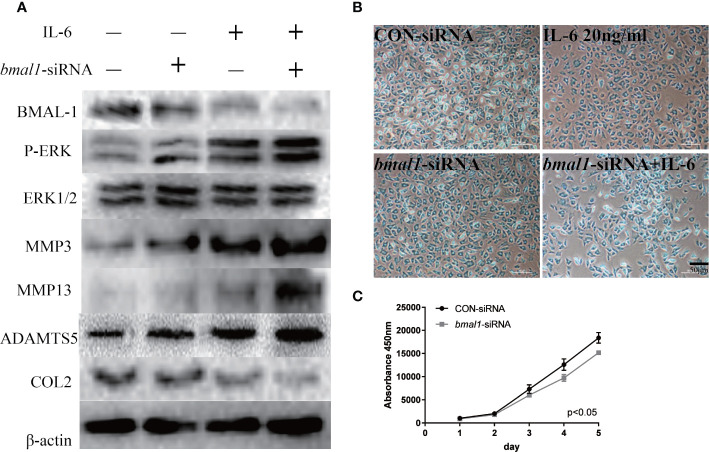
BMAL-1 regulates TMJ-OA *in vitro via* the MAPK/ERK signaling pathway. **(A)** MCCs transiently transfected with control siRNA (con-siRNA) or *bmal1*-siRNAs were cultured in the presence of IL-6. Western blots of MCC lysates showed increased P-ERK, MMP3, MMP9, and MMP13 levels and inhibited COL2 expression in cells transfected with *bmal1*-siRNAs and treated with or without IL-6. **(B)** Alcian blue staining results for MCCs transfected with 50 nM con-siRNA or 50 nM *bmal1*-siRNAs in normal medium or stimulated with 20 ng/ml IL-6 for 12 h. **(C)** BMAL-1 knockdown inhibited MCC proliferation in normal medium, as determined by CCK-8 assays. All experiments were performed in triplicate, and the results are expressed as the mean ± SD. The P value is written in the figure corner. Scale bar: 50 μm.

To continue exploring the regulatory relationship between BMAL-1 and P-ERK, *bmal1*-siRNA was added to rat MCCs to reduce BMAL-1 expression. We next detected P-ERK/ERK/MMP3/13/ADAMTS5/COL2 expression in the presence or absence of IL-6. Western blotting showed that P-ERK expression was upregulated, but total ERK expression was unchanged, indicating that BMAL-1 inhibition led to the activation of ERK phosphorylation (**Figure 5A**). MMP3/13/ADAMTS5 expression was upregulated, and COL2 was inhibited by *bmal1*-siRNA with or without IL-6. In addition, Alcian blue staining and CCK-8 assays showed that *bmal1*-siRNA inhibited chondrocyte proliferation and cartilage matrix protein expression (**Figures 5B, C**).

**Figure 6:**
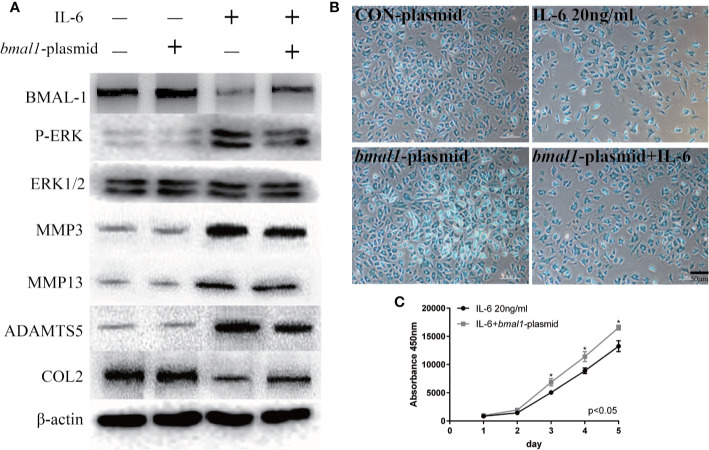
IL-6-treated chondrocytes increase matrix degradation inhibited by BMAL-1. **(A)** MCCs transiently transfected with control plasmid or *bmal1* plasmid were cultured in the presence of IL-6. Western blotting showed that IL-6-induced increases in P-ERK, MMP3, MMP9, and MMP13 were abrogated in BMAL-1-overexpressing cells, and IL-6-induced decreases in COL2 were reversed by the *bmal1* plasmid. **(B)** Alcian blue staining results for MCCs transfected with 20 nM con plasmid or 20 nM *bmal1* plasmid and stimulated with 20 ng/ml IL-6 for 12 h. **(C)** BMAL-1 overexpression enhanced MCC proliferation in medium containing 20 ng/ml IL-6, as determined by CCK-8 assays. All experiments were performed in triplicate, and the results are expressed as the mean ± SD. The P value is written in the figure corner. Scale bar: 50 mm.*P < 0.05.

In contrast, the *bmal1* plasmid inhibited P-ERK expression when chondrocytes were stimulated with IL-6, as shown in **Figure 6A**. Similarly, IL-6-induced MMP3/13 and ADAMTS5 upregulation was abrogated by BMAL-1 overexpression. Meanwhile, COL2 expression decreased in IL-6-stimulated rat MCCs, and these changes were reversed in cells transfected with *bmal1* plasmid. Images and a graph of the Alcian blue staining results are presented in **Figure 6B**. The data show that BMAL-1 overexpression significantly promoted cartilage matrix protein production. Transfection of the *bmal1* plasmid inhibited the effects of IL-6 on chondrocyte proliferation (**Figure 6C**). We then investigated whether BMAL-1 alone modulated P-ERK, MMP3/13, and ADAMTS5 expression, and the inhibitory effect was amplified after IL-6 was added.

### BMAL-1 Overexpression Reverses TMJ-OA in Rats

**Figure 7:**
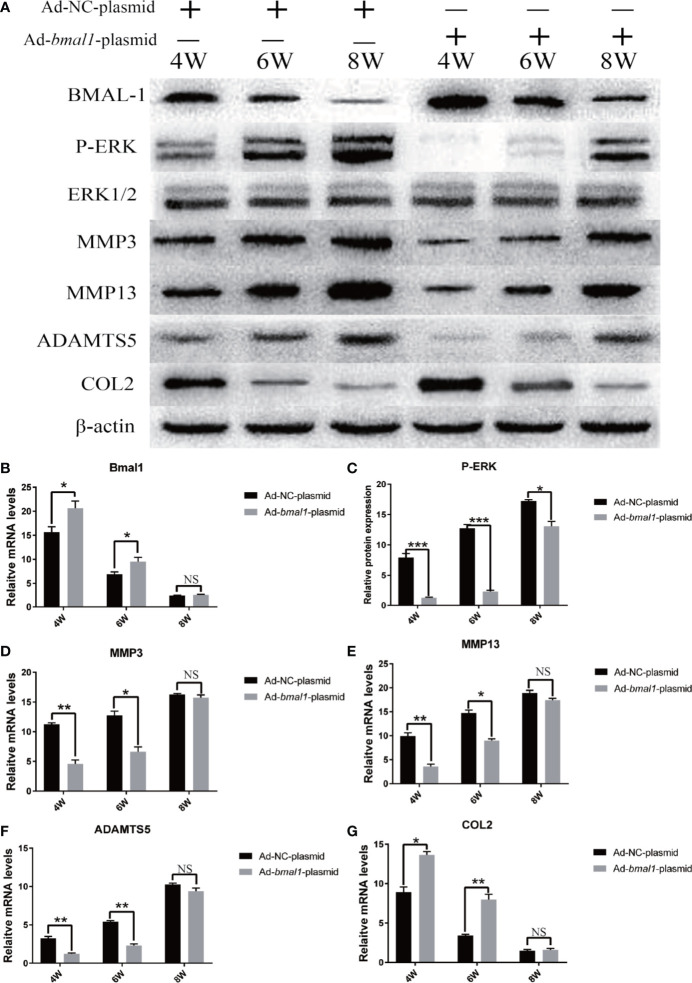
BMAL-1 overexpression reverses TMJ-OA in rats. Ad-NC plasmid and Ad-bmal1 plasmid were injected IA into the TMJs of mice once per week for 4, 6, and 8 weeks. **(A)** Compared with that in rats transfected with Ad-NC-plasmid, the P-ERK, MMP3/13 and ADAMTS5 upregulation was significantly delayed in bmal1 overexpression rats. **(B)** qRT-PCR results indicated decreased *bmal1* mRNA levels *in vivo* after Ad-*bmal1* plasmid transfection. **(C)** Western blotting assay images show that CRD-induced P-ERK upregulation in cartilage was markedly abrogated in *bmal1* overexpression rats. **(D)** The CRD-induced MMP3 mRNA upregulation in cartilage was markedly abrogated in *bmal1* overexpression rats. **(E)** The CRD-induced MMP13 mRNA upregulation in cartilage was markedly abrogated in *bmal1* overexpression rats. **(F)** The CRD-induced ADAMTS5 mRNA upregulation in cartilage was markedly abrogated in *bmal1* overexpression rats. **(G)** The CRD-induced COL2 mRNA downregulation in cartilage was markedly abrogated in *bmal1* overexpression rats. All experiments were performed in triplicate, and the results are expressed as the mean ± SD. *P < 0.05; **P < 0.01, ***P < 0.005. NS, not significant.

To further investigate the function of BMAL-1 in OA pathogenesis, we used *bmal1*-overexpressing rats as a protection-of-function approach (**Figures 7A, B**). Compared with that in rats transfected with the Ad-NC-plasmid, the P-ERK, MMP3/13 and ADAMTS5 upregulation was significantly delayed in *bmal1* overexpression rats (**Figures 7A, C**). Similarly, the downregulation of COL2 expression was significantly delayed. We demonstrated that BMAL-1 overexpression has an inhibitory effect on the TMJ-OA process. Consistent with this, CRD-induced upregulation of MMP3/13/ADAMTS5/COL2 mRNA in the cartilage was markedly abrogated in *bmal1* overexpression rats (**Figures 7D–G**). These studies may provide new insights into the mechanism of BMAL-1 in the balance between cartilage synthesis and catabolism, as well as in the complex pathophysiological mechanism of TMJ-OA, and potentially provide a new method for the prevention and treatment of this disease.

## Discussion

In healthy TMJ cartilage, the periodic regulation of biological rhythm keeps the ECM in a slow and continuous turnover state, commonly known as “homeostasis”, a balance between the anabolic and catabolic activities of matrix synthesis and degradation (Yang and Meng, 2016). Circadian rhythms control the physiological functions of organisms throughout the day, and disturbances in circadian rhythms can cause heart rate irregularities and premature aging in animals (Streuli and Meng, 2019). At the same time, osteoarthritis is one of the characteristics of premature aging in organisms and is affected by circadian disturbances (Berenbaum and Meng, 2016). Vascularization is one of the main features of osteoarthritis and is regulated by P-ERK and ERRγ (Zhao et al., 2019). Vascular and nerve growth are linked through common pathways, which involve the release of angiogenic factors such as vascular endothelial growth factor, β-nerve growth factor and neuropeptide (Mapp and Walsh, 2012; Jahanban-Esfahlan et al., 2018).

Although several studies have shown that chondrocytes have an autonomous circadian rhythm, the process of endochondral ossification is regulated by specific circadian gene products expressed by chondrocytes during postnatal bone formation. However, the effects of circadian disturbances on cartilage homeostasis have not yet been determined (Kato et al., 2006). In this study, we found that disrupting circadian rhythmicity can lead to rhythmic expression profiles of the clock gene *bmal1*, causing upregulated inflammatory factor IL-6 expression and enhanced ERK phosphorylation. Our *in vivo* experiments show that there is a direct correlation between the expression of BMAL-1 and p-ERK. Our experiment disrupted the biological rhythm of rats through an improved sleep deprivation platform, which resulted in deregulated expression of the *bmal1* gene and enhanced phosphorylation of ERK, thus upregulating IL-6 expression and inducing ECM degradation.

In an *in vitro* study, *bmal1*-siRNA transfection caused a marked increase in P-ERK expression. Silencing Bmal1 aggravated the effects of IL-6 on chondrocytes as assessed by the expression of genes such as MMPs, ADAMTS5 and COL2. In contrast, P-ERK expression in chondrocytes decreased after adding the *bmal1* plasmid. However, U0126, which is a recognized ERK inhibitor, reduced the cartilage ECM degradation caused by IL-6 but did not significantly change the BMAL-1 expression level.

Consistent with our findings, the literature has revealed the relationship between the MAPK/ERK signaling pathway and biological rhythm, further extending these results to the other two major members of the MAPK signal family: the JNK pathway and the p38 pathway (Ma et al., 2014; Mehana et al., 2019). These three kinases are rhythmically phosphorylated under normal 24-h circadian rhythm. Another study reported the role of MAPK/ERK signaling pathway in regulating autonomic circadian rhythms and the light-dark initiation of these rhythms, and suggested that Elk-1 represents a new molecular component of the light-induced pathway in the suprachiasmatic nucleus of the hypothalamus of mammals (Coogan and Piggins, 2003).

The relationship between circadian rhythm and osteoarthritis has been confirmed by many studies. For example, a study defined a regulatory mechanism that links the clock gene BMAL-1 in chondrocytes to the maintenance of cartilage homeostasis, and suggests that circadian rhythm disturbance is one of the important risk factors for osteoarthritis (Dudek et al., 2016). B. Guo et al. found that clock gene expression was disturbed in isolated chondrocytes after exposure to pro-inflammatory cytokines (e.g., interleukin-1β, tumor necrosis factor alpha) common to the arthritic process (Guo et al., 2015). Moreover, a recent study has shown that circadian rhythm disturbance is partly related to inflammation and cell autonomous, because synovial fibroblasts in rheumatoid arthritis show changes in the circadian expression of several clock genes after circadian rhythm disturbance, and interfere with the production of circadian rhythms of IL-6 and IL-1 β (Kouri et al., 2013). OA, characterized by articular cartilage breakdown in synovial joints, has long been viewed as the result of “wear and tear”. Our previous studies have confirmed that CRD activates MAPK/ERK signaling pathway and increases the expression of downstream enzymes related to cartilage matrix synthesis and metabolism in rat temporomandibular joint (Ma et al., 2014).

In conclusion, these findings appear to show that CRD may lead to rhythmic gene expression dysregulation, which further leads to MAPK/ERK signaling pathway activation and then aggravates TMJ-OA. In this sense, maintaining the stable expression of rhythmic genes and restoring rhythmic repair would be a reasonable strategy to treat TMJ-OA. Importantly, we showed that the inflammatory cytokine IL-6 is controlled by BMAL-1, and *bmal1* plasmid treatment reduced arthritis in CRD mice; not only P-ERK but also matrix-degrading MMP-3, MMP-13, and ADAMTS5 were significantly reduced in the TMJ. These studies may provide new insights into the clock gene mechanism of endochondral homeostasis and the complex pathophysiological mechanism of TMJ-OA, and may provide new methods for the prevention and treatment of the disease.

## Data Availability Statement

The raw data supporting the conclusions of this article will be made available by the authors, without undue reservation, to any qualified researcher.

## Ethics Statement

The animal study was reviewed and approved by Institutional Animal Care Committee of Shandong University (protocol GR2018017).

## Author Contributions

Conceived and designed the experiments: GKC, HQZ, and CM. Performed the experiments: GKC, HMZ, SXM, LC. Analyzed the data: GKC and JZ. Contributed reagents, materials, and analysis tools: GKC, HMZ, and YZ. All authors contributed to the article and approved the submitted version.

## Funding 

This work was supported by National Natural Science Foundation of China (61771290, 61871393), the Science and Technology Development Plans of Shandong province (Grant 2018GSF118196), Taishan Scholars (tsqn201812137), China Postdoctoral Science Foundation (No. 2019M652408) and Jinan Science and Technology Plan(No.201907098).

## Conflict of Interest

The authors declare that the research was conducted in the absence of any commercial or financial relationships that could be construed as a potential conflict of interest.
