# Corrigendum: Circadian rhythm protein Bmal1 modulates cartilage gene expression in temporomandibular joint osteoarthritis via the MAPK/ERK pathway

**DOI:** 10.3389/fphar.2022.971840

**Published:** 2022-09-08

**Authors:** Guokun Chen, Haoming Zhao, Shixing Ma, Lei Chen, Gaoyi Wu, Yong Zhu, Jie Zhu, Chuan Ma, Huaqiang Zhao

**Affiliations:** ^1^ Department of Oral and Maxillofacial Surgery, School and Hospital of Stomatology, Cheeloo College of Medicine, Shandong University and Shandong Key Laboratory of Oral Tissue Regeneration and Shandong Engineering Laboratory for Dental Materials and Oral Tissue Regeneration, Jinan, China; ^2^ Department of Orthodontics, School and Hospital of Stomatology, Cheeloo College of Medicine, Shandong University and Shandong Key Laboratory of Oral Tissue Regeneration and Shandong Engineering Laboratory for Dental Materials and Oral Tissue Regeneration, Jinan, China; ^3^ Department of Plastic Surgery, Jinan Airong Plastic Surgery Hospital, Jinan, China

**Keywords:** circadian rhythm disturbance, osteoarthritis, temporomandibular joint, BMAL-1, cartilage, interleukin-6, MAPK/ERK pathway

In the published article, there was an error in Figures 3, 4 as published. In Figure 3, the Western blot result of IL-6 was selected incorrectly. In Figure 4, images from **Figure 5B** were accidentally repeated in Figure 4B. The corrected Figures 3, 4 appear below.

**FIGURE 3:**
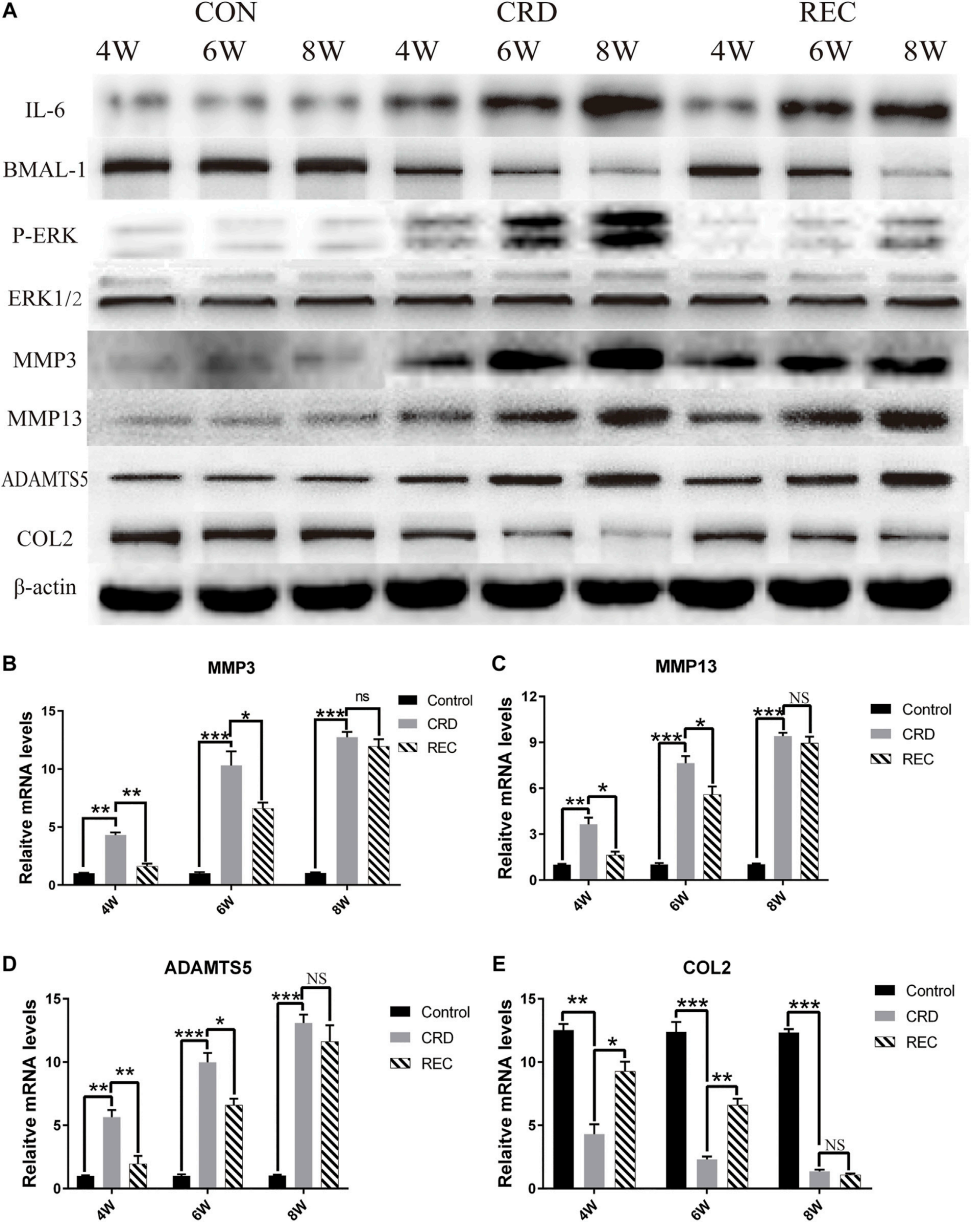
Expression trends for IL-6/P-ERK/BMAL-1/MMP3/MMP13/ADAMTS5/COL2 in the CON, CRD, and REC groups. **(A)** Western blot results for IL-6/P-ERK/BMAL-1/MMP3/MMP13/ADAMTS5/COL2. **(B–E)** RT-qPCR analysis of MMP3/MMP13/ADAMTS5/COL2. All experiments were performed in triplicate, and the results are expressed as the mean ± SD. *p < 0.05; **p < 0.01, ***p < 0.005. NS, not significant.

**FIGURE 4:**
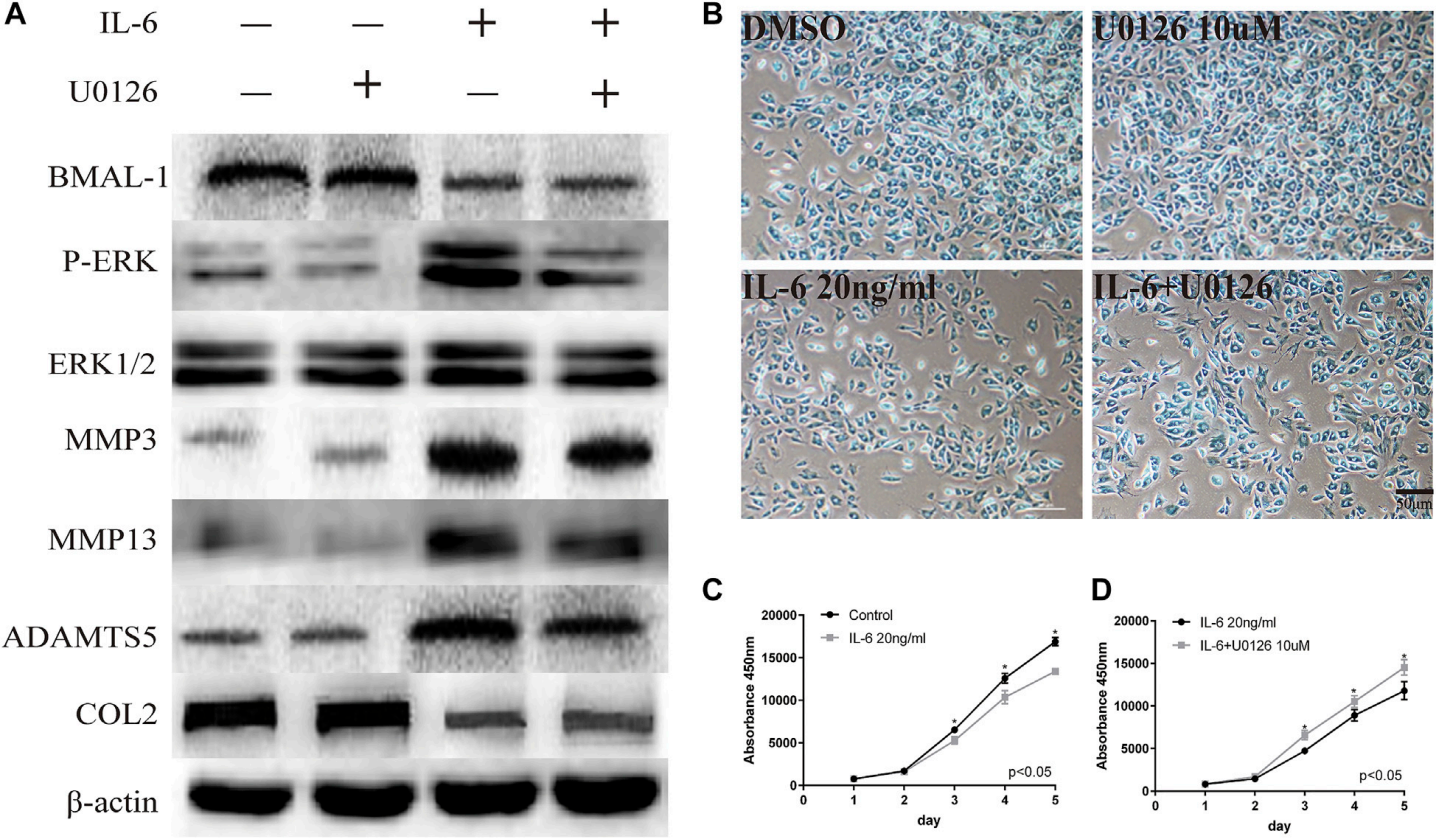
Regulatory relationship between BMAL-1/P-ERK and IL-6. **(A)** Western blot assays demonstrate the relationship between BMAL-1, P-ERK, MMP3, MMP9, MMP13, and COL2, while the expression levels of ERK1/2 and β-actin were the same in the cartilage tissues of the control group, CRD group and REC group. **(B)** Alcian blue staining results for MCCs stimulated with 20 nM or 10 μM U0126 and 20 ng/ml IL-6 for 12 h. **(C,D)** A CCK-8 assay was used to examine the cell proliferation of each group and the effects of U0126; the absorbance was measured at 450 nm. All experiments were performed in triplicate, and the results are expressed as the mean ± SD. The *p* value is written in the figure corner. Scale bar: 50 mm. *p < 0.05.

The authors apologize for this error and state that this does not change the scientific conclusions of the article in any way. The original article has been updated.

