# Supplementary material for: Circadian Rhythm Protein Bmal1 Modulates Cartilage Gene Expression in Temporomandibular Joint Osteoarthritis via the MAPK/ERK Pathway
Source: Front Pharmacol. 2020 Sep 18;11:527744. doi: 10.3389/fphar.2020.527744 (PMC7530270; doi:10.3389/fphar.2020.527744)
Supplement: Supplementary file 1 [file Table_1.docx]

Supplement Table 1:Primers used for RT-PCR analysis in this study

| Genes | Upstream (5’-3’) | Downstream (5’-3’) |
| --- | --- | --- |
| β-actin | CGGTCAGGTCATCACTAT | TGTTGGCATAGAGGTCTT |
| Bmal1 | CTATCTTCCTCGGACACTGC | CTTCTTGCCTCCTGGAGAAG |
| MMP3 | GATGAACGATGGACAGAT | GCTACACATTGGTAAGGT |
| MMP13 | ATGTGGAGTGCCTGATGTG | AAGCGTGTGCCAGAAGAC |
| ADAMTS5 | CGCTGTGATTGAAGATGATG | TGCTGGTAAGGATTGAAGAC |
| col2α1 | AAGAGCAAGGAGAAGAAG | TTACAGTGGTAGGTGATG |
| IL-6 | ACAACCACGGCCTTCCCTA | TCATTTCCACGATTTCCCAGA |
